# Proceed with Caution Before Assigning “Red Flags” in Residency Applications

**DOI:** 10.5811/westjem.2018.2.37960

**Published:** 2018-05-15

**Authors:** Shellie Asher, Kimberly A. Kilby

**Affiliations:** * Albany Medical College, Department of Emergency Medicine, Albany, New York; † Albany Medical College, Department of Family and Community Medicine, Albany, New York

To the Editor:

We read with interest the paper by Bohrer-Clancy et al.^1^ regarding variables in applications to emergency medicine residency that correlate with “adverse outcomes” in training programs. We have some concerns regarding the methods of this paper, and therefore the validity and generalizability of its results.

Inclusion of “extension of residency” as an isolated “adverse outcome” is problematic. These residents were not placed on formal remediation, nor did they fail to complete the residency. Is the extension of residency training for non-academic reasons an “adverse outcome” that should be avoided, or should residency programs and institutions provide a supportive environment such that residents who need additional time due to personal, medical, or family reasons can receive the support they need in order to finish successfully and go on to productive careers? This is the central tenet behind the ACGME’s (Accreditation Council for Graduate Medical Education) Next Accreditation System, which places clinical competency and educational outcomes before program length. In addition, it is unclear with which domains or competencies residents with adverse outcomes had difficulties. Ability to predict issues with medical knowledge, communication, or professionalism may be an important distinction to make depending on the resources of the program to address these issues.

Another concern is the inclusion of a leave of absence (LOA) for any reason as an indicator of potential difficulty. While some reasons for LOA may portend future challenges in medical training, all LOAs are not created equal. It is the responsibility of student advisors to make recommendations regarding LOAs during medical school, and to attach a stigma to any LOA may pressure students to make decisions that are not in their best interest for fear that it will impact their chances of successfully matching.

Although some of these limitations are addressed in the paper, program directors may not have the time or inclination to dive into the details of the study and may take the results at face value, thereby unfairly disadvantaging students who may have taken a LOA for a variety of legitimate reasons during medical school. As educators with a responsibility for providing support, guidance and accountability in medical education, we must not claim that we want students and physicians to achieve educational milestones and also cultivate their own wellness, and then penalize them and future applicants for taking steps to do so.

With respect,

Shellie Asher, MD, MS

Kimberly A. Kilby, MD, MPH
