# Supplementary material for: Proceed with Caution Before Assigning “Red Flags” in Residency Applications
Source: West J Emerg Med. 2018 May 15;19(4):737–8. doi: 10.5811/westjem.2018.2.37960 (PMC6040902; doi:10.5811/westjem.2018.2.37960)
Supplement: Related texts

# Reply: “Proceed with Caution Before Assigning ‘Red Flags’ in Residency Applications”

**Published:** 2018-05-15

**Authors:** Jesse Bohrer-Clancy, Shawn London

**Affiliations:** * University of Connecticut, Department of Emergency Medicine, Farmington, Connecticut; † Hartford Hospital, Department of Emergency Medicine, Hartford, Connecticut

To the Editor:

We would like to thank the Editor for the opportunity to respond to the thoughtful comments from Drs. Asher and Kilby.

Residents who have delayed graduation from residency for non-academic reasons such as the birth of a child, bereavement, and medical reasons were not included in this cohort since while their graduation date might shift, there was no modification of the curriculum on their behalf. We considered an extension of residency to be a situation in which the curriculum was modified with additional clinical time to address one or more deficits in the Accreditation Council for Graduate Medical Education (ACGME) Core Competencies. While the respondents point out that ACGME has introduced the notion of variable progression through residency curriculum with the Next Accreditation System, it is clear that stakeholders in the medical community do not yet accept the concept of a fluid duration of training as a routine matter given the significant amount of additional documentation and justification required from medical licensing boards and hospital credentialing committees. Any substantial change in the residency curriculum or duration of training most certainly involves the disciplinary process of the program and the residency’s Graduate Medical Education Committee. These actions, even when accepted by trainees without the potential for multiple layers of appeals or legal action, are highly time-consuming and stressful for residency faculty and staff. As such the authors reiterate that an extension of residency training represents a negative outcome for a resident.

We recognize that there are many reasons for students to take a leave of absence, all of which we assume to be necessary and appropriate. As in the application of the conclusions of a clinical trial to the care of a specific patient, we expect program faculty to seek the “big picture” in assessment of a specific applicant, not to substitute the findings of our study for good judgment about the likelihood of success for an applicant. In formulating this study the authors did not seek to assign a value judgment or to stigmatize any of the factors examined in this study; rather we were seeking to identify any potential patterns in the overwhelming sea of data available to program faculty in the residency application process.

Most sincerely,

Jesse Bohrer-Clancy, MD

Shawn London, MD
